# Novel Solid-State Potentiometric Sensors Using Polyaniline (PANI) as A Solid-Contact Transducer for Flucarbazone Herbicide Assessment

**DOI:** 10.3390/polym11111796

**Published:** 2019-11-01

**Authors:** Ayman H. Kamel, Abd El-Galil E. Amr, Nashwa S. Abdalla, Mohamed El-Naggar, Mohamed A. Al-Omar, Hamad M. Alkahtani, Ahmed Y. A. Sayed

**Affiliations:** 1 Department of Chemistry, Faculty of Science, Ain Shams University, 11566 Abbasia, Cairo, Egypt; ahkamel76@sci.asu.edu.eg (A.H.K.); anoooosh311@gmail.com (N.S.A.); 2 Pharmaceutical Chemistry Department, Drug Exploration & Development Chair (DEDC), College of Pharmacy, King Saud University, Riyadh 11451, Saudi Arabia; malomar1@ksu.edu.sa; 3 Applied Organic Chemistry Department, National Research Center, 12622 Dokki, Giza, Egypt; 4 Chemistry Department, Faculty of Sciences, University of Sharjah, Sharjah 27272, UAE; m5elnaggar@yahoo.com; 5 Pharmaceutical Chemistry Department, College of Pharmacy, King Saud University, Riyadh 11451, Saudi Arabia; ahamad@ksu.edu.sa (H.M.A.); ahmedyahia009@gmail.com (A.Y.A.S.)

**Keywords:** Solid-contact ion-selective electrode, conducting polymer polyaniline (PANI), flucarbazone herbicide, molecularly imprinted polymers (MIPs)

## Abstract

Novel potentiometric solid-contact ion-selective electrodes (SC/ISEs) based on molecularly imprinted polymers (MIPs) as sensory carriers (MIP/PANI/ISE) were prepared and characterized as potentiometric sensors for flucarbazone herbicide anion. However, aliquat S 336 was also studied as a charged carrier in the fabrication of Aliquat/PANI/ISEs for flucarbazone monitoring. The polyaniline (PANI) film was inserted between the ion-sensing membrane (ISM) and the electronic conductor glassy carbon substrate (GC). The sensors showed a noticeable response towards flucarbazone anions with slopes of −45.5 ± 1.3 (r^2^ = 0.9998) and −56.3 ± 1.5 (r^2^ = 0.9977) mV/decade over the range of 10^−2^–10^−5^, 10^−2^–10^−4^ M and detection limits of 5.8 × 10^−6^ and 8.5 × 10^−6^ M for MIP/PANI/ISE and Aliguat/PANI/ISE, respectively. The selectivity and long-term potential stability of all presented ISEs were investigated. The short-term potential and electrode capacitances were studied and evaluated using chronopotentiometry and electrochemical impedance spectrometry (EIS). The proposed ISEs were introduced for the direct measurement of flucarbazone herbicide in different soil samples sprayed with flucarbazone herbicide. The results agree well with the results obtained using the standard liquid chromatographic method (HPLC).

## 1. Introduction

Pesticides are necessary and widely used in agriculture to resist the harmful effects caused by pests, weeds or diseases that could destroy crops. Furthermore, they enhance the quality of the food produced [1]. However, controlling the residues of these pesticides in foods is a growing source of concern because of their potential risk to the environment and organisms [2]. Flucarbazone-sodium (FLU) is the one that belongs to the sulfonylurea herbicides. It can be used for controlling most dicotyledons weeds in wheat crops. It provides excellent control of wild oats and green foxtail in spring wheat and durum, including weed populations that are resistant to herbicide groups [3]. The maximum residue limit (MRL) of flucarbazone-sodium was set by the US at 0.01 mg/kg in wheat grain and 0.05 mg/kg in wheat straw [4]. Flucarbazone residue is known as the flucarbazone form in addition to its N-desmethyl metabolite in wheat in the year 2006 [5].

To check the existence level and the transfer behavior of FLU after use, it is important to find a reliable analytical method to assess this compound. Some methods were reported in the literature for the determination of flucarbazone, such as liquid chromatography/mass spectrometry (LC-MS/MS) [6], gas chromatography [7], high-performance liquid chromatography (HPLC) [8] and bioassay methods [9,10,11]. For herbicide residue assays in soil samples, the plant assay and chemical methods are frequently used. Despite that the chemical methods are characterized by their specificity and sensitivity, they are expensive, time-consuming, need sophisticated instruments and sample pre-treatment is required. Plant bioassays suffer from different issues such as non-specificity, time-consuming and the non-availability of a true control sample. Besides, different parameters are frequently assessed in plant bioassays.

Ion-selective electrodes (ISEs) are currently attracting considerable attention because of their enhanced selectivity and their ability to detect low concentrations. Further, ISEs have promising applicability for in-situ monitoring due to their quick response time [12] and they find wide applications in environmental [13,14,15], clinical [16,17,18] and pharmaceutical [19,20,21] analyses due to their simplicity, accuracy and selectivity. To date, no single sensor has been described for the determination of flucarbazone.

The development of all-solid-state ion-selective electrodes (SC/ISEs) represent a considerable recent improvement [22,23]. Solid-state ion-selective electrodes are characterized by their lower resistivity and ease of miniaturization due to the elimination of the internal reference solution. They revealed enhanced performance characteristics which make them possible to be integrated into long-term continuous in-situ monitoring in the water environment. Molecularly imprinted polymers (MIPs) became a very important part in the preparation of artificial and strong recognition materials [24,25,26]. The sensors based on MIPs were prepared by assembling the MIP beads onto the surface of the transducer. Therefore, the analyte binding is transformed into a measurable signal. MIPs have been coupled to different techniques such as electrochemical sensors, fluorimetry, piezoelectricity and surface plasmon resonance [27,28,29,30,31,32]. Further, MIPs bring several advantages when integrated with potentiometric sensors for pharmaceutical [17,20,21], clinical [33,34] and environmental analyses [15,23,35,36,37], especially enhanced selectivity for analytes in different matrices.

Polyaniline (PANI) can be considered as one of the most studied conducting polymers (CPs). It is characterized by its cost-effectiveness, high stability and ease of preparation [38]. Ion-selective electrodes (ISEs), based on CPs, can be considered as new types of analytical tools. Further, PANI was successfully introduced in the development of solid-contact ISEs as a solid contact between the electronic conductor and the ion-selective membrane (ISM). The surface characterizations of PANI films, such as the hydrophilicity [39] and surface morphology [40,41], can enhance the properties of the various matrices, in particular, the traditional PVC membranes, and make them more useful in the field of analytical chemistry. So, the deposition of a thin film of PANI as a conducting polymer has been considered in this work in the design of ISEs for flucarbazone herbicide determination.

This work introduces novel solid-contact ion-selective electrodes for flucarbazone determination. The sensors are based on the use of molecularly imprinted polymers (MIPs) or aliquat S 336 as sensory carriers and polyaniline (PANI) as an ion-to electron transducer. The proposed ISEs revealed an enhanced sensitivity and reasonable selectivity for flucarbazone monitoring in different soil samples. These proposed ISEs offered different advantages, such as the ease of designing, the short time required for measurements, high precision and accuracy, high-potential stability and a low limit of detection.

## 2. Materials and Methods

### 2.1. Chemicals and Reagents

Flucarbazone-sodium (99.0% purity) was supplied by Arysta Lifescience Co., Ltd., Yantai, China. Cyromazine, bispyribac sodium, diquate dibromide, and dinotefuran, were purchased from Dr. Ehrenstorfer GmCbH (Berlin, Germany). Polyaniline (emeraldine salt) (Average *M*_w_ > 15,000, 3–100 μm particle size), methacrylic acid (MAA), ethylene glycol dimethacrylate (EGDMA), benzoyl peroxide (BPO), tetra-dodecyl ammonium tetrakis (4-chlorophenyl) borate (ETH 500), aliquat 336 and acetonitrile were purchased from Sigma-Aldrich Inc. (St. Louis, MO, USA). High molecular weight poly (vinyl chloride) (PVC), dioctyl phthalate (DOP) and tetrahydrofuran (THF) were obtained from Fluka AG (Buchs, Switzerland).

A 10^−2^ M flucarbazone solution was prepared by dissolving the appropriate amount of the pure form of the pesticide in 100 mL of distilled water and the pH of the solution was adjusted to pH 7 using 30 mM phosphate buffer solution (PBS). The dilutions of FLU (10^−3^–10^−7^ M) were prepared in 30 mM PBS, pH 7.

### 2.2. MIPs Synthesis

The imprinted polymer beads were synthesized using the precipitation polymerization method. In brief, 1.0 mmol of FLU as a template molecule was mixed with 3.0 mmol MAA monomer and 3.0 mmol of EGDMA as a cross-linking reagent. All were dissolved in 25 mL acetonitrile as a porgenic solvent. In addition to 80.0 mg of BPO (initiator), the reaction mixture was added to a 25-mL glass-capped bottle successively. The removal of the dissolved oxygen was carried out after purging the solution with a flow of N_2_ gas for 10 min. Solution homogenization for the solution was obtained by sonication for 15 min. The polymerization process was carried out after adding the glass-capped bottle in an oil bath for 18 h at 80 °C. Using the Soxhlet extraction, the template was removed using NaOH/methanol (1:9, *v/v*). The resulting polymer beads were washed with methanol and then, they were left to dry overnight at room temperature. In the absence of flucarbazone and under similar conditions, the non-imprinted polymer (NIP) was also synthesized.

### 2.3. Electrode Fabrication

Glassy carbon electrodes (GCEs) (4 mm diameter) were sonicated for 15 min in de-ionized water then cleaned with ethanol for further 15 min. The electrodes were left for 1 h to dry. A 10-µL of 2 mg/mL PANI and 3 mg of ETH 500 (all are dissolved in THF) were applied via drop- casting on the conductive glassy carbon substrate. A layer of the conducting polymer was formed after drying with a thickness close to 0.25 µm. The composition of the sensing membrane was prepared by dissolving 100 mg of components in 1.0 mL THF: Sensor I [MIP or NIP (6.0 wt %), PVC (33.2 wt %), and DOP (60.8 wt %)] and sensor II [aliquat 336 (2.5 wt %), PVC (33.2 wt %), and DOP (64.3 wt %)], respectively. The membrane cocktail (100 μL) was drop-casted onto the dried PANI layer and left to dry for 2 h. The GC/FLU-ISEs without PANI were prepared in the same way. All GC/FLU-ISEs were firstly conditioned in 1 × 10^−3^ M FLU solution for 1 day and then in 1 × 10^−6^ M FLU for another day.

### 2.4. Electromotive Force (EMF) Measurements

The EMF of the constructed cell was measured at 25 ± 1 °C using pH/mV meter (Orion 720/SA, Cambridge, MA, USA). In a stirred FLU solution, the potentiometric measurements were performed after immersing the designed electrodes in conjunction with the reference electrode in this solution. To eliminate the liquid-junction potential, a correction was made for the EMF readings using the Henderson equation. The Debye-Huckel approximation was utilized for activity estimation of the working standard FLU solutions. According to the IUPAC recommendations, the performance features of the proposed electrodes were evaluated [42,43]. All experiments were performed with at least three electrodes.

### 2.5. Electrochemical Characterization

The potential drift for the proposed sensors was checked using electrochemical impedance spectroscopy (EIS) and chronopotentiometry measurements. Metrohom potentiostat/galvanostat (Autolab, model 204) purchased from Metrohom Instruments (Herisau, Switzerland) was used for these measurements. The proposed electrodes acted as working electrodes and immersed in 10^−2^ M FLU solution in conjunction with Ag/AgCl/KCl (3M) single junction reference electrode (Model 6.0733.100, Metrohm), and an auxiliary electrode made from a Pt wire. For chronopotentiometry, a constant current of ±1 nA was introduced on the working electrode for 60 s followed by a reversed current for another 60 s. The EIS measurements were carried out through the frequency range from 10^4^ to 0.1 Hz. The amplitude of the sinusoidal excitation signal was 100 mV.

### 2.6. Applications to Real Samples

The designed ISEs were introduced to test their applicability in detecting the concentration of FLU inside the real samples. The samples containing FLU were soil samples collected from different agricultural lands sprayed with flucarbazone pesticide. A 250 g of different soil samples was soaked into 250 mL water and sonicated for 2 h. After filtration, the obtained filtrate was then analyzed by the proposed potentiometric cell and the results were compared to those obtained by the standard liquid chromatography/mass spectrometry (LC-MS/MS) method [6]. In brief, high-performance liquid chromatography (HPLC) (Agilent 6410, USA) coupled with a triple quadrupole mass spectrometer (Agilent, Santa Clara, CA, USA) and an electrospray ionization (ESI) source. ZORBAX SB-C18 column (50 × 2.1 mm, 1.8 μm; Agilent, USA) were used for the chromatographic separation. The chromatographic conditions are: 0.3 mL/min flow rate; mobile phase is acetonitrile and water (0.1% HCOOH) (*v/v* 70:30); isocratic mode and the sample injection volume is 5 μL.

## 3. Results and Discussions

### 3.1. Characterization of MIP Beads

**Figure 1:**
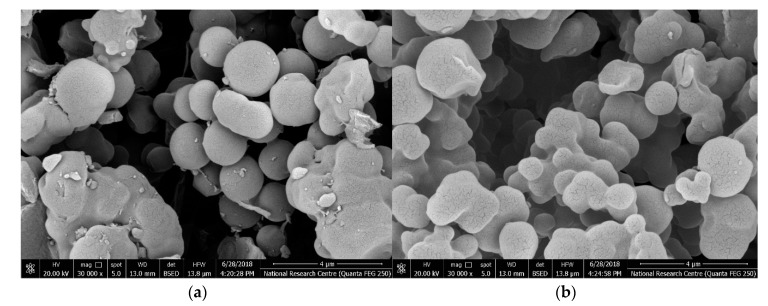
SEM images of (**a**) molecularly imprinted polymers (MIP) and (**b**) non-imprinted polymer (NIP) nanobeads.

**Figure 2:**
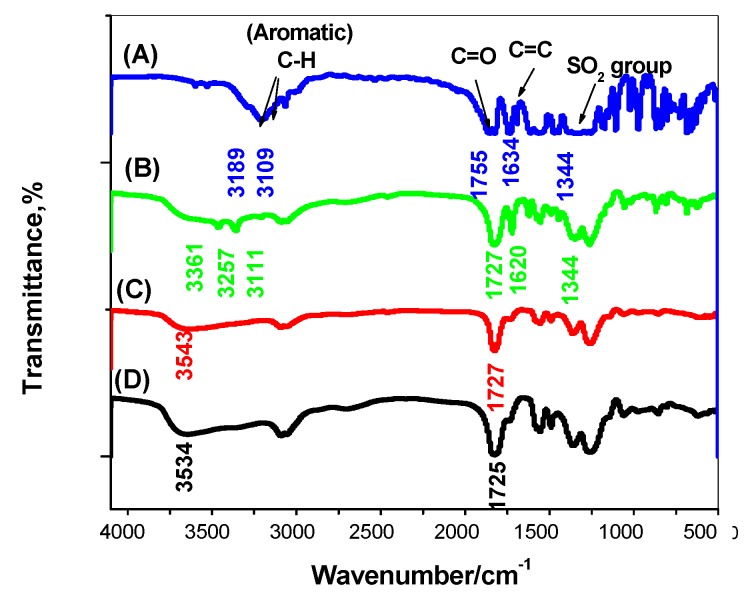
FT-IR spectra for (**A**) flucarbazone (FLU), (**B**) non-washed MIP; (**C**) washed MIP; (**D**) NIP beads.

The morphology of the prepared flucarbazone imprinted beads was examined using the scanning electron microscope (SEM) [JOEL-JSM-IT500HR, Osaka, Japan]. As presented in Figure 1a, the molecularly imprinted particles (MIP) were of spherical and uniform-size with a diameter distribution of 0.9–1.2 µm. Therefore, the beads are well dispersed in the sensor membrane inducing more available binding sites in the sensing membrane. Additionally, it will reduce the resistance of the membrane. A similar morphological structure and particle size distribution as in MIP particles were obtained for NIP nano-beads (Figure 2b).

The Fourier-transform infrared spectroscopy (FT-IR) measurements were carried out using the FT-IR spectrometer (Alpha II, Bruker ABCO, Cairo, Egypt) using the attenuated total reflection (ATR) technique. As shown in Figure 2, the FTIR spectra of flucarbazone, non-washed MIP, washed/MIP, and NIP beads were examined. As shown in Figure 2a, a υC–H stretch vibration peak appeared at 3189 and 3109 cm^−1^ assigned for aliphatic and aromatic C–H, respectively. The carbonyl group (C=O) and aromatic (C=C) appeared at 1755 and 1634 cm^−1^, respectively. The medium and sharp peak at 1344 and 1200 cm^−1^ appeared and assigned for the SO_2_ group. Further, almost all of these peaks already present in the FT-IR spectrum of the non-washed MIP were either in the same position or shifted (Figure 2b). This can be considered as proof for the successful imprinting of flucarbazone. As shown in Figure 2c, the washed/MIP exhibited an O–H peak at 3543 cm^−1.^ This is very close to the peak assigned to the O–H present in NIP beads (3534 cm^−1^) (Figure 2d). From the above-mentioned data, the imprinting of flucarbazone using MAA was carried out successfully. Nonetheless, the complete removal of flucarbazone from the backbone of MIP was achieved.

### 3.2. Potentiometric Performance of the All-Solid-State ISEs

**Figure 3:**
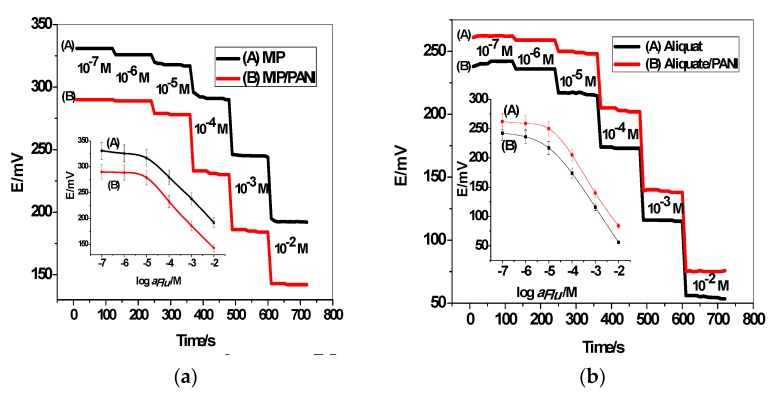
The time-dependent potential response curve of the proposed electrodes integrated with (**a**) MIP nanobeads curve and (**b**) aliquat.

The potentiometric responses of the proposed electrodes GC/PANI-MIP/FLU^−^-ISE and GC/PANI-Aliquat/FLU^−^-ISE to 10^−8^–10^−2^ M flucarbazone were tested. As shown in Figure 3, the time-dependent potential response curve of the proposed electrodes in the presence and absence of a PANI layer revealed a stable potential response and the response time was <5 s. For the sensors based on MIP beads, they showed Nernstian response with slopes of −41.7 ± 2.2 (n = 5, r^2^ = 0.9988) and -45.5 ± 1.3 mV/decade (n = 5, r^2^ = 0.9998) in the linearity range of 10^−5^ –10^−2^ M and detection limits of 7.4 × 10^−6^ and 5.8 × 10^−6^ for GC/MIP/FLU^−^-ISE and GC/PANI-MIP/FLU^−^-ISE, respectively. The NIP nano-beads were tested as a control. The sensors based on NIP beads revealed a worse response performance towards FLU than compared with those based on MIP beads under the same conditions. The sensor exhibited an anionic slope of −17.3 ± 0.9 (r^2^ = 0.9976) mV/decade over a linear range of 1.0 × 10^−^^2^–1.0 × 10^−^^3^ M with a detection limit of 7.5 × 10^−^^4^ M. This confirms the enhanced specificity of the MIP binding sites in the membrane and flucarbazone ion. The sensors based on aliquat in the absence and presence of PANI revealed Nernstian response with slopes of −54.1 ± 1.1 (n = 5, r^2^ = 0.9972) and −56.3 ± 1.5 mV/decade (n = 5, r^2^ = 0.9977) in the linearity range of 5.0 × 10^−5^–10^−2^ and 3.0 × 10^−5^–10^−2^ M and detection limits of 1.7 × 10^−5^ and 8.5 × 10^−6^ for GC/Aliquat/FLU^−^-ISE and GC/PANI-Aliquat/FLU^−^-ISE, respectively. All potentiometric characteristics are shown in Table 1.

### 3.3. Potential Stability

**Figure 4:**
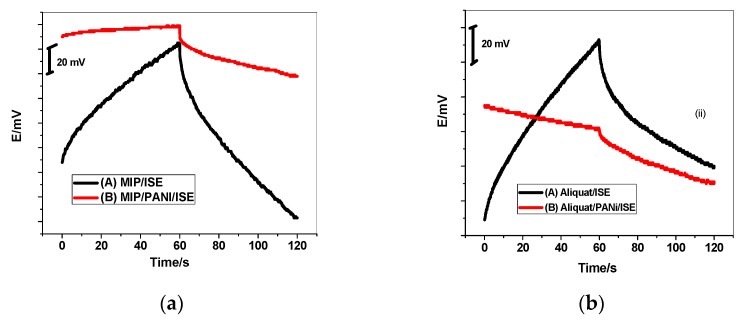
Chronopotentiograms for (**a**) MIP and (**b**) Aliquat based sensors recorded in 10^−3^ M.

By current-reversal chronopotentiometry, the potential stability of flucarbazone ISEs were evaluated (Figure 4). The potential drift of the GC/PANI-MIP/FLU^−^-ISE and GC/PANI-aliquat/ FLU^−^-ISE obtained from the slope (Δ*E*/Δ*t*) of the curve is 11.6 and 39.3 µV/s. It was found that these values are much lower than those obtained from the GC/MIP/FLU^−^-ISE (146 µV/s) and GC/aliquat/FLU^−^-ISE (69.5 µV/s). These results indicate that the potential stability of all proposed ISEs is enhanced by introducing the PANI layer as the solid contact. According to the equation, Δ*E*/Δ*t = I*/*C*_L_ proposed by Bobacka [44], the low-frequency capacitance (*C*_L_) for GC/PANI-MIP/ FLU^−^-ISE and GC/PANI-aliquat/FLU^−^-ISE was calculated to be 86.2 ± 2.3 and 25.4 ± 0.9 µF, respectively. (Δ*E*/Δ*t* is the potential drift, *I* is the applied current and *C_L_* is a double layer or low-frequency capacitance). These values are lower compared with the GC/MIP/FLU^−^-ISE (6.8 µF) and GC/aliquat/FLU^−^-ISE (14.4 µF). This can be explained based on the existence of the ion-sensing membrane on the PANI film [44].

**Figure 5:**
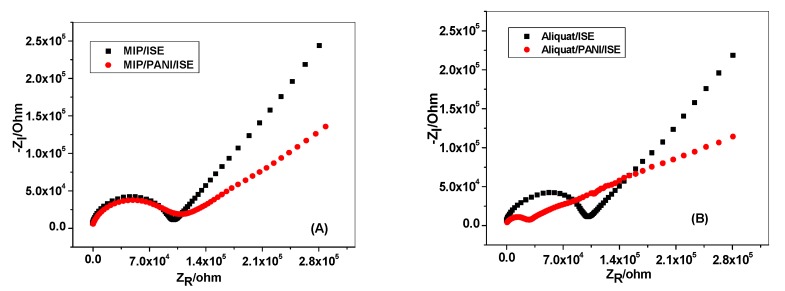
Impedance spectra of the (**A**) MIP; and (**B**) Aliquat based sensors recorded in 10^−3^ M flucarbazone-sodium.

The impedance spectra of flucarbazone ISEs measured in 10^−3^ M flucarbazone-sodium covering the frequency range from 10^4^ to 0.1 Hz is shown in Figure 5. It can be seen that all proposed electrodes revealed a high-frequency semicircle, which is attributed to both the resistance of the membrane (*R_b_*) and the charge transfer resistance (*R_CT_*) [45]. The high-frequency resistance for the GC/PANI-MIP/FLU^−^-ISE and GC/PANI-Aliquat/FLU^−^-ISE was measured and it was found that the (*R_CT_*) between the ion-sensing membrane and the GC substrate decreases upon applying the PANI layer as a transducer. Furthermore, the GC/PANI-MIP/FLU^−^-ISE and GC/PANI-Aliquat/FLU^−^-ISE have a small low-frequency semicircle in comparison to the GC/MIP/FLU^−^-ISE and GC/Aliquat/FLU^−^-ISE, which has a large low-frequency semicircle. This can be attributed to the presence of a PANI layer which alters the interfacial conduction between the ion-sensing membrane and the GC substrate. Therefore, it enhances the double-layer capacitance (*C_L_*) of the ion-selective electrode. The double layer capacitance (*C_L_*) for GC/PANI-MIP/FLU^−^-ISE and GC/PANI- Aliquat/FLU^−^-ISE was *C_L_* = 11.7 ± 0.7 µF and *C_L_* = 37.7 ± 1.2 µF, respectively, as compared with that of the GC/MIP/FLU^−^-ISE (6.5 µF) and GC/Aliquat/FLU^−^-ISE (7.3 µF).

### 3.4. Water Layer Test

**Figure 6:**
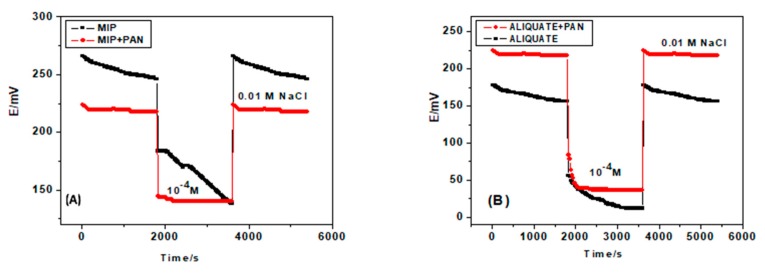
Water layer tests for flucarbazone ISEs based on (**A**) MIP; and (**B**) Aliquat.

The water film formation between the ion-sensing membrane and the electronic conductor substrate could cause the potential drift for the solid-state ion-selective electrode. A procedure has been proposed for the water-layer test by Fibbioli et al. [46]. The potential responses of the GC/PANI-MIP/FLU^−^-ISE and GC/PANI-Aliquat/FLU^−^-ISE in addition to their correspondence without the PANI layer, GC/MIP/FLU^−^-ISE and GC/Aliquat/FLU^−^-ISE, were sequentially measured in 0.01 M NaCl for 1/2 h, 10^−4^ M flucarbazone-sodium for 1/2 h and then back in 0.01 M NaCl. Figure 6 shows that the sensors have PANI as a solid-contact layer which revealed a much more stable response than those that had no PANI layer. There are no positive or negative drifts in the potential when changing the ISE from NaCl to flucarbazone-sodium, and again to NaCl. This is a piece of clear evidence for the non-existence of the water layer between the ion-sensing membrane and the electronic conductor GC. In addition, it reflects the high hydrophobicity of the PANI layer.

### 3.5. Selectivity

The selectivity of the proposed sensors was carried out and the selectivity coefficient values (Log *K^pot^_i,j_*) were calculated using the modified separate solution method reported in [47]. The values of the calculated selectivity coefficients are presented in Table 2. All the proposed ISEs revealed excellent selectivity towards flucarbazone over diquate, cyromazine, dinotefuran, bispyribac and other common anions. GC/PANI-Aliquat/FLU^—^ISE revealed better selectivity over diquate, cyromazine, dinotefuran, bispyribac, SO_4_^2−^, NO_3_^−^, CH_3_COO^−^ and CN^−^ than GC/PANI-MIP/FLU^—^ISE. From the results presented in Table 2, an enhanced selectivity for these presented ISEs can be successfully applied for the trace-level determination of flucarbazone in different matrices.

### 3.6. Flucarbazone Assessment in Real Samples

The application of the proposed ISEs to flucarbazone assessment in soil samples collected from different agricultural lands was completed. Three different soil samples were collected after 10 days of spraying the herbicide. Before the measurements, the sensors were firstly calibrated, Then, the same batch of sensors was utilized directly in the analysis of the sample. The results obtained by the proposed ISEs agreed fairly well with those obtained by the standard liquid chromatography/Mass spectrometry (LC-MS/MS) method (Table 3). It can be noticed that the proposed flucarbazone-ISEs have promising feasibility for the assessment of flucarbazone herbicide in environmental samples.

## 4. Conclusions

In this work, polyanline (PANI) was utilized successfully as an effective ion-to-electron transducer for developing solid-contact flucarbazone-ISEs. PANI revealed unique features such as a high interfacial area, high double-layer capacitance and enhanced conductivity with high hydrophobicity. Therefore, the proposed ISEs exhibited a stable potentiometric response in the range of 10^−^^5^ to 10^−^^2^ M and 10^−4^–10^−2^ M with the detection limit of 5.8 × 10^−6^ and 8.5 × 10^−6^ M for GC/PANI-MIP/FLU^—^ISE and GC/PANI-Aliquat/FLU^—^ISE, respectively. The sensors introduced great acceptable features such as a fast response, high selectivity, reliable precision, possible automation, and a satisfactory accuracy for real sample analysis.

## Figures and Tables

**Table 1:** Potentiometric characteristics of flucarbazone ion-selective electrodes (ISEs) in 30 mM phosphate buffer solution (PBS), pH 7.

Parameter	GC/MIP/FLU^−^-ISE	GC/PANI-MIP/FLU^−^-ISE	GC/Aliquat/FLU^−^-ISE	GC/PANI-Aliquat/FLU^−^-ISE
Slope (mV/decade)	−41.7 ± 2.2	−45.5 ± 1.3	−54.1 ± 1.1	−56.3 ± 1.5
Correlation coefficient (r^2^)	0.9988	0.9998	0.9972	0.9977
Linear range (M)	10^−2^–10^−5^	10^−2^–10^−5^	10^−2^–5 × 10^−5^	10^−2^–3 × 10^−5^
Detection limit (M)	7.4 × 10^−6^	5.8 × 10^−6^	1.7 × 10^−5^	8.5 × 10^−6^
Working pH range (pH)	4–9	4–9	4–9	4–9
Time response (s)	<10	<10	<10	<10
Accuracy (%)	98.6	99.1	99.2	99.5
Precision (%)	1.1	0.9	0.7	0.8
Standard deviation (mV%)	1.1	1.3	0.9	1.1

**Table 2:** Selectivity coefficient values for the proposed flucarbazone ISEs.

Interfering ion, *J*	(Log *K^pot^_i,j_*)*	
	GC/PANI-MIP/FLU^−^-ISE	GC/PANI-Aliquat/FLU^−^-ISE
Diquat	−3.55 ± 0.07	−3.62 ± 0.02
Cyromazine	−2.95 ± 0.08	−4.21 ± 0.06
Bispyribac	−2.42 ± 0.08	−4.54 ± 0.07
Dinotefuran	−3.43 ± 0.06	−4.36 ± 0.03
Cl^−^	−3.47 ± 0.01	−4.58 ± 0.03
Br^−^	−3.79 ± 0.02	−3.38 ± 0.04
SO_4_^2−^	−3.45 ± 0.09	−4.93 ± 0.07
NO_3_^−^	−3.62 ± 0.02	−3.79 ± 0.08
CH_3_COO^−^	−3.19 ± 0.06	−5.10 ± 0.09
CN^−^	−2.09 ± 0.05	−3.58 ± 0.03

* Mean value obtained from three corresponding pairs of concentrations of flucarbazone and the respective interfering anion in the Nernstian response range ± standard deviation.

**Table 3:** Potentiomeric determination of flucarbazone in different soil samples.

Sample	Amount of Flucarbazone (µg/g)		
	GC/PANI-MIP/FLU^−^-ISE	GC/PANI-Aliquat/FLU^−^-ISE	(LC-MS/MS) Method [6] *
**1**	8.5 ± 0.3	8.1 ± 0.4	7.8 ± 0.1
**2**	15.6 ± 0.7	14.7 ± 0.7	15.2 ± 0.4
**3**	12.3 ± 0.9	11.8 ± 0.6	11.1 ± 0.2

* Average of five measurements ± standard deviation.
